# Exploring the *Trypanosoma brucei* Hsp83 Potential as a Target for Structure Guided Drug Design

**DOI:** 10.1371/journal.pntd.0002492

**Published:** 2013-10-17

**Authors:** Juan Carlos Pizarro, Tanya Hills, Guillermo Senisterra, Amy K. Wernimont, Claire Mackenzie, Neil R. Norcross, Michael A. J. Ferguson, Paul G. Wyatt, Ian H. Gilbert, Raymond Hui

**Affiliations:** 1 The Structural Genomics Consortium (SGC), University of Toronto, Toronto, Ontario, Canada; 2 Department of Tropical Medicine, School of Public Health and Tropical Medicine, Tulane University, New Orleans, Louisiana, United States of America; 3 Division of Biological Chemistry and Drug Discovery, College of Life Sciences, University of Dundee, Dundee, Scotland, United Kingdom; Northeastern University, United States of America

## Abstract

Human African trypanosomiasis is a neglected parasitic disease that is fatal if untreated. The current drugs available to eliminate the causative agent *Trypanosoma brucei* have multiple liabilities, including toxicity, increasing problems due to treatment failure and limited efficacy. There are two approaches to discover novel antimicrobial drugs - whole-cell screening and target-based discovery. In the latter case, there is a need to identify and validate novel drug targets in Trypanosoma parasites. The heat shock proteins (Hsp), while best known as cancer targets with a number of drug candidates in clinical development, are a family of emerging targets for infectious diseases. In this paper, we report the exploration of *T. brucei* Hsp83 – a homolog of human Hsp90 – as a drug target using multiple biophysical and biochemical techniques. Our approach included the characterization of the chemical sensitivity of the parasitic chaperone against a library of known Hsp90 inhibitors by means of differential scanning fluorimetry (DSF). Several compounds identified by this screening procedure were further studied using isothermal titration calorimetry (ITC) and X-ray crystallography, as well as tested in parasite growth inhibitions assays. These experiments led us to the identification of a benzamide derivative compound capable of interacting with *Tb*Hsp83 more strongly than with its human homologs and structural rationalization of this selectivity. The results highlight the opportunities created by subtle structural differences to develop new series of compounds to selectively target the *Trypanosoma brucei* chaperone and effectively kill the sleeping sickness parasite.

## Author Summary

Sleeping sickness, or human African trypanosomiasis (HAT), is a deadly neglected disease for which new therapeutic options are badly needed. Current drugs have several liabilities including toxicity and route of administration limiting their efficacy to combat the disease. Our study aimed at validating a potential new drug target against *Trypanosoma brucei*, its heat shock protein 83 (Hsp83). The chaperone was screened against a repurposed library composed of inhibitors against the human Hsp90. The compounds were assayed in their ability to bind the *T. brucei* protein and to kill the parasite. Our work has identified selective and high-affinity chemical compounds targeting the parasitic Hsp83. Additionally, structural studies were conducted to explore the observed selectivity of selected inhibitors. Our work has validated *T. brucei* Hsp83 as a potential target for future drug discovery campaigns. It has also shown the strength of repurposing chemical libraries developed against human proteins, emphasizing the possibility to piggyback current and past drug discovery efforts for other diseases in the search for new drugs against neglected tropical diseases.

## Introduction

Human African trypanosomiasis (HAT), better known as sleeping sickness is a vector-borne disease present in sub-Saharan Africa, transmitted by *Glossina* tsetse flies and caused by the protozoan parasite *Trypanosoma brucei* [1]. Two subspecies of this kinetoplastid parasite cause disease in humans and are present with different geographic distributions, *T. b. gambiense* and *T. b. rhodesiense*. The former is responsible for chronic disease in Western and Central Africa, while infection by the latter leads to the acute form present in Eastern and Southern Africa. During the initial stage of the infection, the *Trypanosoma* parasite lives and multiplies in the blood and tissue fluids of its human host, thanks to an elaborate mechanism for evading the host immune system. The parasite then invades the central nervous system (CNS) to give rise to the fatal stage 2 infection, during which the classic clinical symptoms of HAT occur. Currently, there are five clinically used treatments, which are prescribed based on the causative species and the stage of the disease [2], [3], [4]; however, the toxicity of existing drugs and inappropriate route of administration limit the efficacy of the current chemotherapy. Consequently, HAT is one of the most neglected tropical diseases due to the limited availability of safe and cost-effective control tools [1], [2]. New methods to treat patients are needed to treat and to eventually eliminate the disease. Among the potential new drug targets, molecular chaperones represent an interesting group already validated in other disease areas. Furthermore, a large number of inhibitors are already available [5], several of which have been proved to be effective anti-proliferatives against several parasites *in vitro* [6], [7], [8], [9], [10].

Among the chaperones, heat shock protein 90 (Hsp90) – alternately referred to as Hsp83 or Hsp86 because of the variable molecular weight amongst different orthologues – is a hallmark of the stress response at the cellular level, and a major component of the eukaryotic proteome [11]. Its main function is to serve as a molecular chaperone helping nascent polypeptides avoid mis-folding and limiting protein aggregation during thermal stress [12]. In eukaryotes, key regulatory proteins require Hsp90 assistance as a part of their maturation process and are known as Hsp90 clients [11]. Not surprisingly, limiting Hsp90 chaperone function either by genetic or chemical means has been found to result in a net reduction of functional client proteins within the cell. This severely impacts cell survival and homeostasis, not to mention its ability to cope with environmental stress [11]. Several of the client proteins in humans are over-expressed in cancer cells. Consequently, development of inhibitors against human Hsp90 (hereafter referred to as Hsp90) has been a highly active field in the anticancer drug therapy field [5], [13], [14]. These compounds from natural and synthetic sources have been found to be effective anti-proliferatives against cancer cells *in vitro* [15], with over a dozen having advanced into clinical trials [16]. Although the compounds represent diverse chemical scaffolds with unique properties, they all inhibit the enzymatic ATPase function of the chaperone by binding the ATP pocket in the N-terminal domain (NTD), which together with the middle and C-terminal domains define the highly conserved Hsp90 protein architecture [11]. During its catalytic cycle, the Hsp90 serves as a molecular clamp, binding both ATP and client protein at its NTD and middle domains respectively. The transient dimerization of the NTDs is coupled with both the ATP hydrolysis and chaperone activity [17]. While in the resting state, the Hsp90 dimerizes solely via its C-terminal domain. In addition to a multitude of client proteins, the Hsp90 also binds a cohort of co-chaperones that regulate the closing and opening cycle [11], [17].

Like most eukaryotes, all protozoan parasites feature a Hsp90 orthologue in their genomes. *Plasmodium* Hsp90 is essential, with geldanamycin effective at inhibiting parasite growth at sub-micromolar concentrations [18]. In *Trypanosoma* and *Leishmania* parasites, Hsp83 is implicated in thermally induced stage differentiation [19]. *T. brucei* Hsp83 (*Tb*Hsp83), the subject of interest herein, is 59% identical in sequence to human Hsp90α. This sequence identity increases to over 70% in the N-terminal ATPase domain, with notable sequence divergence only in a few short stretches (Fig. S1).

To explore the potential of *Tb*Hsp83 as a drug target, we employed a number of biochemical and biophysical techniques. Our approach began with the heterologous expression of the protein and the characterization of its enzymatic activity. Subsequently, a panel of known Hsp90 inhibitors was screened against the parasitic chaperone using differential scanning fluorimetry (DSF), with the results compared to their binding to the human Hsp90 isoforms. Efficacy of the compounds in inhibiting *T. brucei* growth correlated with the biophysical results. A chemical profile was generated from the screening results, highlighting chemical scaffolds that bind *Tb*Hsp83 more strongly than its human counterparts as well as those that have equal affinity for both. Structural analysis shows the binding modes of some of the more potent ligands, including those effective in killing the parasite in cellular assays, suggesting the possibility of developing anti-trypanosomal drug leads against *Tb*Hsp83.

## Materials and Methods

### Protein cloning, expression and purification

The full-length coding region of *T. brucei* Hsp83 (gene Tb927.10.10980 - TritrypDB, http://www.Tritrypdb.org/ [20]) was cloned from genomic DNA. Full-length Hsp83 protein (Met1 to Asp704) and NTD (Met1 to Lys213) clones were obtained both including an N-terminal His6-tag. The two proteins were expressed and purified as previously described [21]. Briefly, clones were grown in TB media in a LEX bioreactor system (Harbinger Biotechnology and Engineering Corp., Ontario, Canada). Overnight starter cultures were left to grow at 37°C until reaching an OD_600_ value around 5, cooled to 15°C, and subsequently induced overnight with 0.5 mM IPTG. Cells were harvested by centrifugation and the pellets resuspended in 40 ml per liter of culture in 50 mM hepes pH 7.5, 500 mM NaCl, 5 mM imidazole, 5% glycerol, 1 mM benzamidine and 1 mM phenylmethyl sulfonyl fluoride (PMSF), then flash frozen in liquid nitrogen and stored in −80°C until needed.

The re-suspended pellets were pretreated with 0.5% CHAPS and 500 U of benzonase for 40 minutes at room temperature and cells were mechanically lysed using the M-110EH Microfluidizer Processor (Microfluidics Corp., MA, USA). The cell lysate was centrifuged to eliminate cells debris and the cleared lysate was loaded onto a DE52 anion exchange resin (Whatman, MA, USA) followed by a 2 mL Ni-NTA (Qiagen, MD, USA). The Ni-NTA column was then washed with 200 mL of a buffer consisting of 50 mM hepes pH 7.5, 500 mM NaCl, 30 mM imidazole and 5% glycerol. The protein was eluted with 15 mL of a buffer consisting of 50 mM hepes pH 7.5, 500 mM NaCl, 250 mM imidazole and 5% glycerol. Following elution, 1 mM EDTA and 1 mM TCEP were added to the sample. The sample was then incubated overnight with TEV protease at 4°C to cleave the His6-tag in 10 mM hepes, pH 7.5, 500 mM NaCl and 1 mM DTT. The imidazole was adjusted to 15 mM and the sample was loaded onto a pre-equilibrated 2.5 mL Ni-NTA column. The sample was allowed to bind to the nickel resin for 30 minutes after which the flow through containing the tag cleaved protein was collected. The protein was further purified by size exclusion chromatography on a Superdex 200 (GE Healthcare, NJ, USA) in the case of the full length protein or a Superdex 75 in the case of the NTD, both columns equilibrated with a buffer consisting of 10 mM hepes, pH 7.5 and 500 mM NaCl. The full length *Tb*Hsp83 peak fractions eluted at retention volumes consistent with a dimeric enzyme and monomeric for the NTD. The proteins were concentrated in an Amicon Ultra centrifugal filter device (Millipore, MA, USA) and their identities were confirmed by SDS-PAGE and mass spectroscopy.

### ATPase characterization

The malachite green assay was used to characterize the ATPase activity of *T. brucei* Hsp83. We determined the amount of inorganic phosphate produced after incubating 95 nM *Tb*Hsp83 full-length 1 h at 37°C with its nucleotide substrate in 50 mM tris-HCl pH 7.5, 75 mM NaCl and 6 mM MgCl_2_. The substrate was evaluated at a concentration range from 1.9 µM to 2 mM. The kinetic parameters were obtained by fitting initial rate against substrate concentration using a nonlinear regression algorithm (SigmaPlot 2000 software; SPSS Inc., Chicago, USA).

### Compound synthesis

**Figure 1:**
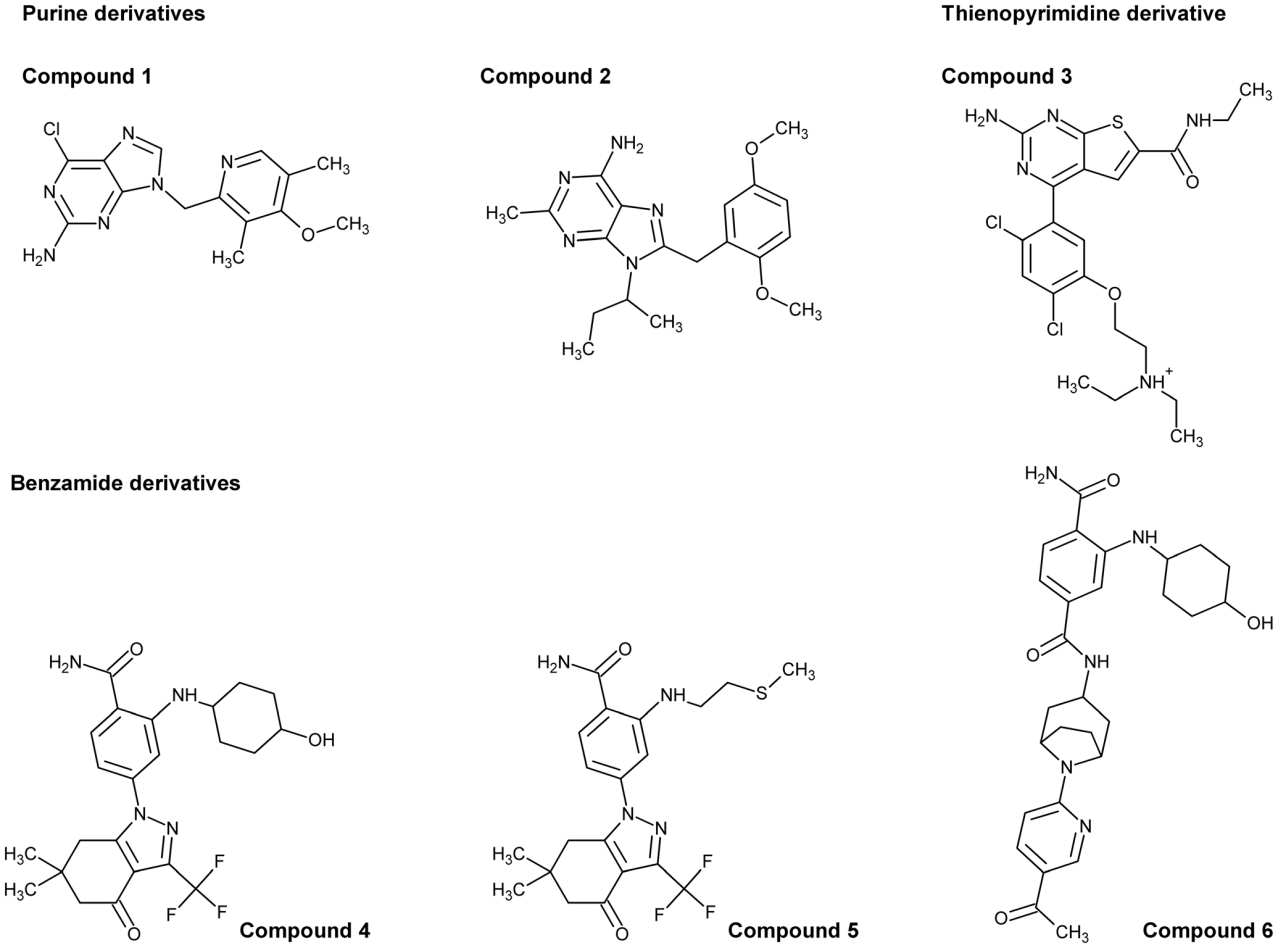
Hsp90 inhibitors. Chemical representation of the compounds co-crystallized with *T. brucei* Hsp83 NTD and the non- commercially available compounds used in this study. All the compounds are listed in the supplementary material (Fig. S2).

Many of the Hsp90 inhibitors used in the assays reported herein were purchased from commercial sources (Supplemental Table S1), with six compounds commercially unavailable (Fig. 1) and synthesized using methods already reported in the literature: compound **1** (6-chloro-9-(4-methoxy-3,-dimethylpyridin-2-ylmethyl)-9H-purin-2-ylamine (BIIB021) [22]; compound **2** (9-(sec-butyl)-8-(2,5-dimethoxybenzyl)-2-methyl-9H-purin-6-amine) [23]; compound **3** – (2-amino-4-[2,4-dichloro-5-(2-diethylaminoethoxy)phenyl]thiopheno[2,3-d]pyrimidine-6-carboxylic acid ethylamide) [24]; compound **4** (4-[6,6-dimethyl-4-oxo-3-(trifluoromethyl)-4,5,6,7-tetrahydro-1H-indazol-1-yl]-2-[(trans-4-hydroxycyclohexyl)amino]benzamide) (SNX-2112) [25]; compound **5** (4-(6,6-dimethyl-4-oxo-3-(trifluoromethyl)-4,5,6,7-tetrahydro-1H-indazol-1-yl)-2-((2-(methylthio)ethyl)amino)benzamide) [25]; compound **6** (N4-[8-(5-acetylpyridin-2-yl)-8-azabicyclo[3.2.1]oct-3-endo-yl]-2-[(trans-4-hydroxycyclohexyl)amino]benzene-1,4-dicarboxamide) [26].

### Differential scanning fluorimetry (DSF)

DSF screening was carried out using a LightCycler 480 Real Time PCR System (Roche Applied Science, Quebec, Canada). The NTDs from *Tb*Hsp83 as well as human Hsp90s α and β were buffered in 100 mM hepes pH 7.5, 150 mM NaCl and assayed in a 384-well format. The final concentration of the protein sample was optimized between 0.05 and 0.2 mg/ml for each NTD (concentration selected based on pre-testing results to avoid saturation of the fluorescence detector). The Hsp90 inhibitors were used at a final concentration of 25 µM (the full list of compounds is available as supplementary information, Fig. S2). SYPRO Orange (Molecular Probes, OR, USA) was added as a fluorescence probe in a dilution of 1∶1000. The experiments were conducted between 20°C to 95°C at a heating rate of 1°C per minute. The recorded fluorescence reads were fitted to the Boltzmann sigmoid function using Bioactive software (Harbinger Biotechnology and Engineering Corp., Ontario, Canada). The inflection point of each fitted curve is defined as the melting temperature (*T*_m_). The observed temperature shift, Δ*T*_m_, was recorded as the difference between *T*_m_ of a sample and a reference in the same plate (i.e. *T*_m_ of the protein with ligand minus *T*_m_ of the protein without ligand). Thermal shifts above 2°C were considered significant.

### ITC measurements

ITC experiments were performed using a VP-ITC instrument (GE healthcare, NJ, USA). Injections of Hsp90 inhibitor solution were added to sample solutions of *Tb*Hsp83 NTD, with concentrations of 200 mM and 20 µM respectively. Titrations were conducted at 20°C in 100 mM hepes pH 7.5, 150 mM NaCl and 0.7% DMSO. The experimental data were fitted to a theoretical titration curve using the software package Origin (OriginLab Corporation, MA, USA), with Δ*H* (binding enthalpy in kcal mol^−1^), *K_a_* (association constant) and *n* (number of binding sites per monomer), as adjustable parameters. *K*_d_ (dissociation constant) was calculated as 1/*K*_a_. The standard free energy change and other thermodynamic parameters were calculated using the equations Δ*G* = −*RT* ln (*K_a_*) and Δ*G* = Δ*H*−*T*Δ*S*, where Δ*G*, Δ*H*, and Δ*S* are the changes in free energy, enthalpy, and entropy of binding, respectively.

### Protein crystallization, crystallographic data collection, structure determination and refinement

A purified sample of the tag-cleaved *Tb*Hsp83 NTD was crystallized using the sitting drop vapor diffusion method in the presence of three different inhibitors (Fig. 1). All crystals were obtained by mixing one part of protein solution at 12 mg/ml (10 mM hepes pH 7.5, 500 mM NaCl, 2 mM TCEP and 4 mM MgCl_2_) containing 2 mM inhibitor with one part of reservoir solution. In the case of compound 1, the reservoir solution contained 2 M ammonium sulfate, 2% PEG400, 100 mM hepes pH 7.5; for the thienopyrimidine derivative compound it contained, 25% PEG 3350, 200 mM ammonium acetate and 100 mM hepes pH 7.5; and for the benzamidine derivative it included, 25% PEG 8000, 200 mM NaCl, 100 mM sodium cacodylate pH 5.5. Crystals appeared within three weeks and were cryo-protected in glycerol supplemented mother liquor before being flash cooled in liquid nitrogen. The diffraction data was collected using an X-ray source equipped with an R-Axis IV detector (Rigaku, TX, USA) and processed with HKL2000. The structures were determined by molecular replacement. The *Leishmania major* Hsp83 NTD (PDB code 3H80) served as a search model for the first complex structure of the *T. brucei* NTD, which was used as the model for subsequent structures. Phaser [27], [28] was used for the molecular replacement calculations, while model building was performed with COOT [29], [30] and the structures were refined with REFMAC5 [31] from the CCP4 suite of programs [32] and Buster-TNT [33]. Inhibitor coordinates and geometry restraints were created within the PRODRG topology server [34]. The stereochemistry of both models was checked by MOLPROBITY [35]. Relevant data collection and refinements statistics are shown in Table 1. The coordinates for the structure and their structure factors have been deposited with the Protein Data Bank (http://www.pdb.org [36]).

**Table 1:** Summary of crystallographic parameters and model refinement statistics for *T. brucei* Hsp83 NTD complexes.

Inhibitor	Compound 1	Compound 3	Compound 4
**Space group**	**P**2_1_	**P**2_1_2_1_2_1_	**P**2_1_2_1_2_1_
**a (Å)**	55.65	60.04	70.92
**b (Å)**	65.81	60.94	72.10
**c (Å)**	62.74	126.99	152.15
**β**	92.3°	-	-
**Resolution range (Å)**	50 – 2.00 (2.03 – 2.00)	35 – 2.15 (2.19 – 2.15)	50 – 2.6 (2.71 – 2.6)
**Unique reflections**	30581 (16406)	25590 (1252)	24806 (2665)
**Redundancy**	3.7 (3.4)	6.7 (6.6)	3.7 (3.6)
**R_merge_ (%)**	7.5 (52.8)	7.1 (72.4)	7.1 (37.1)
**Completeness (%)**	99.3 (93.7)	97.8 (97.1)	99.7 (98.5)
**I/σ(I)**	9.6 (2.4)	12.6 (3.05)	10.4 (2.9)
***V*** **_M_ (Å^3^/Da)**	2.4	2.2	2.5
**PDB code**	3O6O	3OMU	3OPD
**R-value**	0.19 (0.20)	0.22 (0.28)	0.18 (0.23)
**R_free_**	0.23 (0.26)	0.27 (0.38)	0.24 (0.29)
**r.m.s bonds (Å)**	0.010	0.009	0.01
**r.m.s angles (°)**	1.106	1.236	1.19
**Number of atoms**			
**Protein**	3229	3108	4629
**Organic**	54	62	99
**Solvent**	193	96	359
**Ramachandran plot**			
**Favored**	426/432	411/421	588/603
**Outliers**	0/432	0/421	1/603
**Average B factors (Å^2^)**	32.72	41.71	56.35

### Structure analysis

Structure superposition were calculated with LSQKAB [37] as implemented in the CCP4 package. Ligand-protein interactions were calculated and 2-D plots were generated by LigPlot+ [38]. The structure figures herein were generated with PyMOL (DeLano Scientific, Palo Alto, California, USA. http://www.pymol.org).

### Parasite growth inhibition assay

Assay conditions for *T. brucei* 427 cell based assay were followed as previously reported in the literature [39].

## Results

### Enzymatic assays

Kinetic constants of the enzymatic activity of *Tb*Hsp83 were determined using the standard malachite green assay. With *V_max_* = 37±1.6 pmoles P/min (Fig. S3), the ATPase activity of this parasitic chaperone is weak compared with other ATPases such as AAA (ATPases Associated with various cellular Activities) or P-type enzymes, but within the range observed for other Hsp90 proteins [7], [40], [41]. The *K_m_* (360 µM ATP) was also within the range of values previously determined for other Hsp90 chaperones from other organisms (Table 2).

**Table 2:** ATPase kinetic parameters for *T. brucei* Hsp83 and reported activities for other Hsp90s*.

	*T. brucei*	Human Hsp90β [40]	Yeast [41]	*P. falciparum* [7]
*K* **_m_ (µM)**	358±34	840±60	510±70	611
*k* **_cat_ (s^−1^)**	0.12			0.00165
*V* **_max_ (µM P** ***_i_*** ** s^−1^)**	1.13×10^−2^±4×10^−4^			

(^*^) values ± standard deviation.

### Differential scanning fluorimetry (DSF)

**Figure 2:**
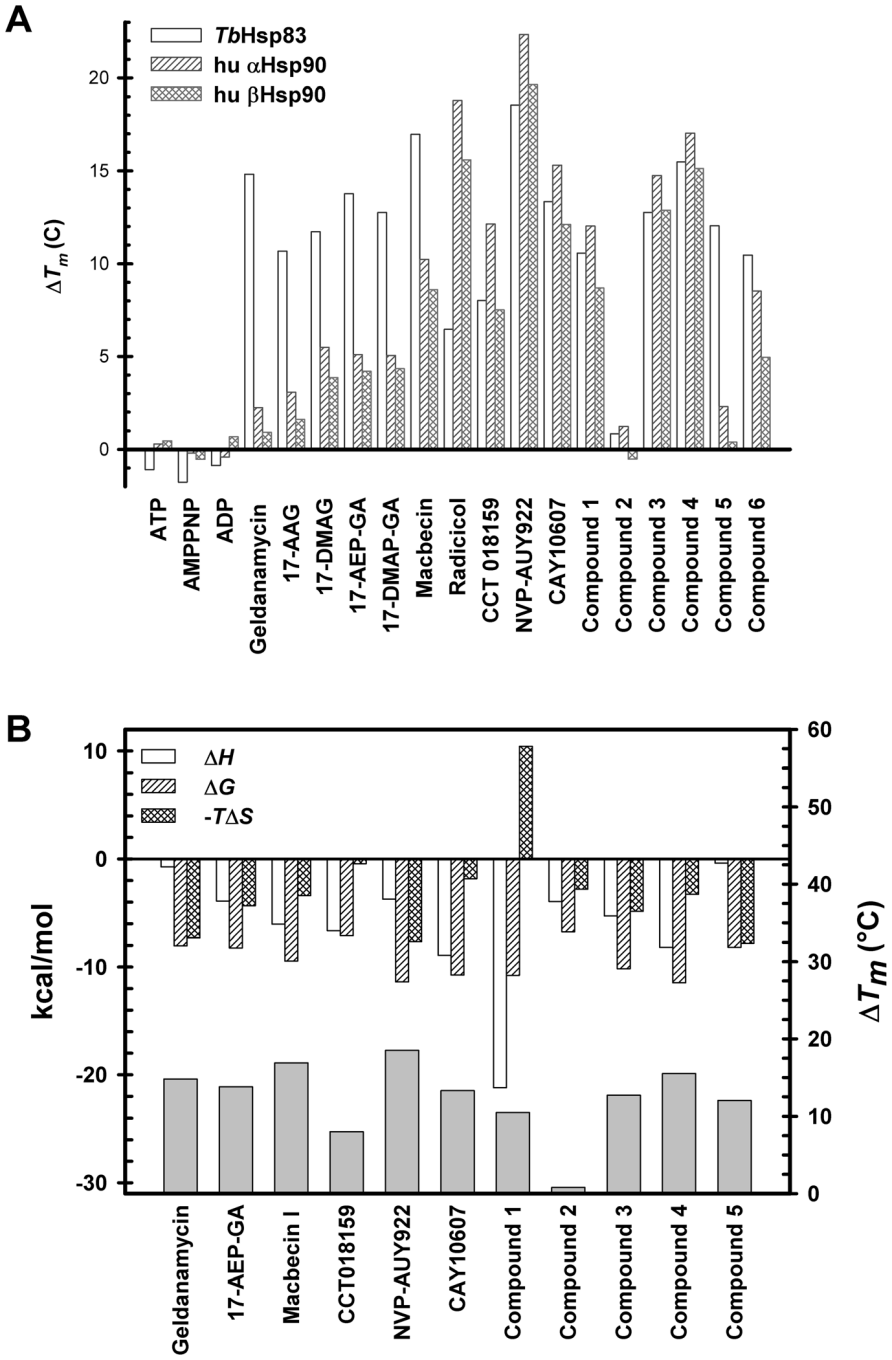
Thermal shift assay results and thermodynamic parameters of *T. brucei* Hsp83 NTD interactions with inhibitors. (A) The Δ*T*_m_ values obtained by DSF are presented in this bar representation for a subset of the Hsp90 inhibitors tested. The DSF results for all compounds tested are available as supplementary material. (B) Binding Interactions Average values for Δ*G*, Δ*H*, -*T*Δ*S* are given for protein-compound interactions measured by ITC. The Δ*T*_m_ values derived from DSF studies are also indicated. The raw ITC graphs are available as a supplementary material.

The DSF assay results for *Tb*Hsp83, Hsp90α and Hsp90β in the presence of known or putative Hsp90 inhibitors are shown in a bar graph for NTD binders (Fig. 2A) and as a heat map for all the compounds tested (Fig. S2). Clearly, a number of compounds bind *Tb*Hsp83 with different levels of affinity, generating a chemical fingerprint (Fig. 2A). Furthermore, screening the human homologues as controls generated different fingerprints indicative of differences in chemical sensitivity between the parasite and human chaperones that might be exploited in the design of selective inhibitors. In particular, macbecin, geldanamycin and derivatives [6], and compound 5 demonstrated markedly stronger binding to *Tb*Hsp83, whereas radicicol and NVP-AUY922 had stronger affinity for human Hsp90. Overall, this experiment demonstrated the potential for designing parasite-specific inhibitors.

### Isothermal titration calorimetry (ITC)

As a secondary assay, some of the compounds were tested with *Tb*Hsp83 using isothermal titration calorimetry. All the interactions measured were exothermic and enthalpy driven (Fig. 2B). Most of them displayed positive entropic contributions, with the exception of the interaction between the parasitic chaperone and compound 1 with a negative entropic term (Δ*S* = −35.6 kcal/mol). This interaction was also significantly more exothermic than the others suggesting an enthalpic compensation. Most of the measured interactions showed high affinity, with *K*_d_ values in the nanomolar range (Table 3), compounds 1 and 4, CAY10607 and NVP-AUY922 displayed sharp transitions curves in the binding enthalpies which could lead to an underestimation of the affinity (Fig. S4). Also the ITC derived affinities could have been affected in other compounds as visualized by poor baseline in the binding thermograms for compounds 2, 4, 5 and geldanamycin; and, molar rations higher than 1 for 17-AEP-GA, Macbecin I and NVP-AUY922. The compromised results were caused by limited solubility of some of the tested compounds and protein precipitation during the ITC experiments. But, the reported values represent the best values obtained from multiple repetitions. Furthermore, the *K*_d_ derived values are in agreement with previously reported affinities even in the case of compromised ITC measurements such as the one reported for NVP-AUY92 (Table 3). The highest affinity recorded for the *Trypanosoma* chaperone was in the low nanomolar range (*K*_d_ = 2.8 nM for a benzamide derivative, compound 4). As stated above, the recorded affinities were similar - within the same order of magnitude - to previously reported ITC measurements of Hsp90 from other species (Table 3) [42], [43], [44], [45], [46], [47]. The only exception was the 2.5 fold higher affinity displayed by *T. brucei* Hsp83 for macbecin I when compared to the *K*_d_ value reported for the yeast counterpart [45]. The ITC experiments generally corroborated and validated the DSF results (Fig. S5), and also provided thermodynamic insights about the interaction between *Tb*Hsp83 and the inhibitors.

**Table 3:** *T. brucei* Hsp83 NTD affinity* and parasite growth inhibition measurements.

	Trypanosoma brucei					Human		Previous ITC measurements				
Compound	Δ*T*_m_ (°C)	*K*_d_ (µM)		S.D.	EC_50_ (µM)	Δ*T*_m_ (°C)		*K*_d_ (µM)		S.D		Ref.
**AMPPNP**	0.3		N.D.					141		―	*b*	[42]
			N.D.					111	±	6.4	*d*	[46]
**AMPPSP**	N.D		N.D.					109	±	42	*c*	[44]
**ADP**	0.1		N.D.			0.3*^a^*	0.5*^b^*	15		―	*b*	[42]
			N.D.					35	±	3.7	*c*	[44]
**Geldanamycin**	14.8	1.000	±	0.246	0.013 [6]	2	4.3	2.9	±	0.32	*d*	[46]
**17-AEP-GA**	13.8	0.714	±	0.034		5.1	4.2					
**17-AAG-GA**	10.7		N.D.		0.038 [6]	3.1	1.6	0.3	±	0.06	*a*	[47]
**17-DAMG-GA**	11.7		N.D.		0.003 [6]	5.5	3.9	0.35	±	0.04	*b*	[42]
**Macbecin I**	16.9	0.093	±	0.010		10.2	8.6	0.24	±	0.03	*d*	[45]
**Radicicol**	6.5		N.D.		0.070 [6]	18.8	15.6	0.00004		―	*a*	[47]
			N.D.					0.00015		―	*b*	[47]
			N.D.					0.025	±	0.0004	*c*	[44]
			N.D.					0.016	±	0.00003	*d*	[46]
**NVP-AUY922**	18.5	0.003	±	0.001		22.3	19.6	0.0017	±	0.0005	*c*	[43]
**CCT018159**	8.01	4.975	±	0.678		12.1	7.5		―			
**CAY10607**	13.3	0.010	±	0.001		15.3	12.1		―			
**Compound 1**	10.5	0.009	±	0.002	0.31	12.0	8.7		―			
**Compound 2**	0.8	9.260	±	3.430	13	1.2	−0.5		―			
**Compound 3**	12.7	0.026	±	0.009	0.21	14.7	12.9		―			
**Compound 4**	15.5	0.003	±	0.001	0.10	17.0	15.1		―			
**Compound 5**	12.03	0.794	±	0.604	0.13	2.3	0.4		―			
**Compound 6**	10.4		N.D.		0.22	8.5	4.9		―			

(^*^)Affinity measurements by ITC and DSF methods are reported.

***a***) Human Hsp90α; ***b***) Human Hsp90β; ***c***) Human TRAP-1; ***d***) Yeast Hsp83. N.D. – not determined.

### Parasite growth inhibition assays

Several of the compounds characterized using DSF and ITC assays were evaluated for their ability to kill *T. brucei* in a continuous *in vitro* culture system. Five out of the six compounds tested showed EC_50_ against the blood stream stage of *T. brucei* 427 in the submicromolar range (Table 3). The outlier compound, namely compound **2**, also showed a non-significant Δ*T*_m_ on *Tb*Hsp83 and the lowest affinity measured by ITC, consistent with correlation between binding affinity and *in vitro* efficacy (Fig. S5), although more data would be needed to corroborate the correlation.

### Crystallography

**Figure 3:**
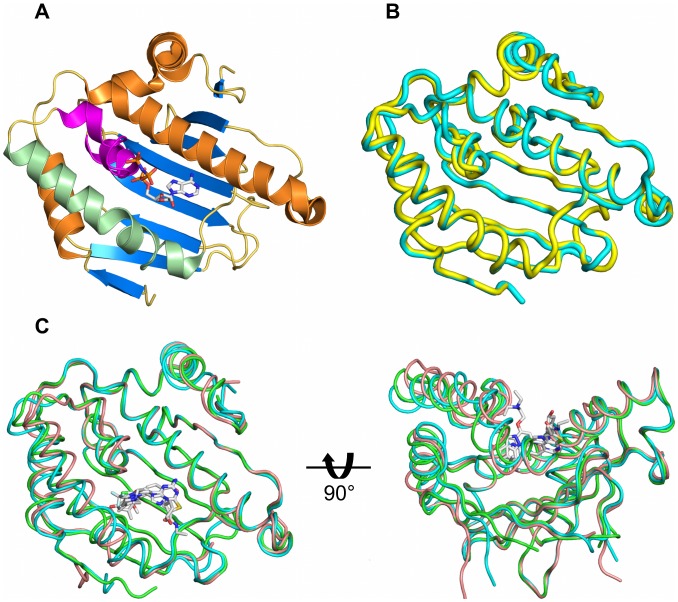
Crystal structure of *T. brucei* Hsp83 NTD. (A) A cartoon representation of the parasitic protein, the nucleotide lid region is highlighted in magenta, α-helices 3 and 4 are shown in light green. The ATP mimetic shown to indicate the position of the nucleotide binding site was derived from the *L. major* complex structure (PBD entry 3H80). (B) NTDs structural overlay between *Tb*Hsp83 (cyan) and human αHsp90 (yellow – PDB entry 3QDD). (C) Structural overlay of *Tb*Hsp83 NTD inhibitor complexes. Two orthogonal views of ribbon representations of the three complexes and the compounds are indicated as sticks. The structures are colored as follows, compound **1** complex – cyan; thienopyrimidine derivative compound **3** – green; benzamidine derivative compound 4 – brown.

**Figure 4:**
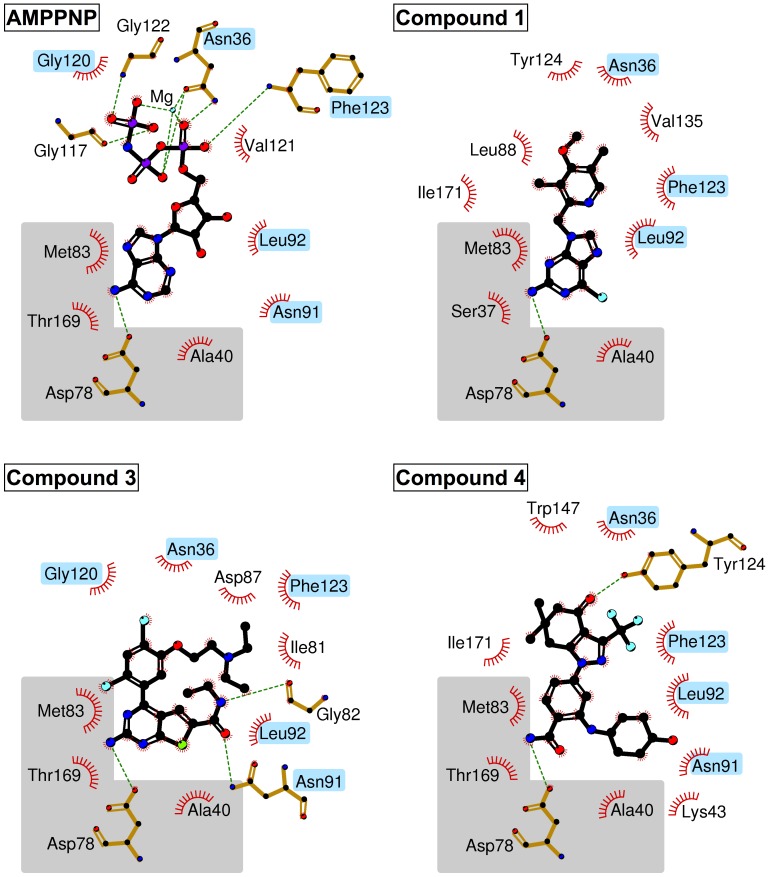
Protein-ligand interaction maps of *T. brucei* Hsp83 NTD complexes. The Hsp83 NTD-AMPPNP interaction map was derived from the *L. major* complex structure (PDB entry 3U67). The gray region represents the adenosine binding pocket; additional residues in common with the nucleotide analogue complex structure are highlighted in blue.

We have crystallized the N-terminal domain of Hsp83 from *T. brucei* in complex with three different synthetic inhibitors – compounds **1**, **3** and **4** (Fig. 3 & 4), but did not succeed in generating crystals of high acceptable diffraction quality in *apo* form or bound with ADP or ATP analogues. In general, the *Tb*Hsp83 crystals obtained were sensitive to the cryo-protection buffer and harvesting techniques. All the complex crystals were obtained by co-crystallization of the parasitic chaperone and the inhibitors. Two out of the three crystals belonged to the same space group but each one had unique cell parameters. Two of the crystals, thienopyrimidine (compound **3**) and benzamide (compound **4**) complexes, belonged to the orthorhombic system, containing two or three non-crystallographic symmetry (NCS) related complexes per asymmetric unit (ASU) respectively. The other crystal (compound **1** complex) was a primitive monoclinical with two copies per ASU. Despite these variations in space groups and cell parameters, no significant differences were observed between the three crystal forms in solvent content (*V*_M_ values in Table 1) and most of the lattice contacts were shared. Extensive crystal contacts included an anti-parallel arrangement of the 1st and last β strands. Additional contacts included the loop regions connecting α helices 1 and 2, and helices 4 and 5.

Overall, the structure of the NTD of *Tb*Hsp83 was similar to previously determined Hsp90 nucleotide-binding domains (Fig. 3). The electron density in all three complexes was well defined for the whole polypeptide chain, facilitating the modeling of almost all of the cloned residues with the exception of the last few at the C-terminus. The final models showed good stereochemistry parameters. The resolution allowed us to explicitly model a significant number of solvent molecules. Also the electron density was unambiguous for the three different inhibitors present in each crystal form and for each molecule in the ASU (Fig. S6). Due to the absence of an *apo Trypanosoma* structure we compared it with a *Leishmania* Hsp90 in complex with AMPPNP and ADP also determined by our group (PDB codes 3H80 and 3U67, Hills *et al.*, unpublished results). The structural differences observed between each complex and this previously determined parasitic Hsp90 were linked to the nature of the inhibitor bound to the nucleotide binding site and not to any *Trypanosomes*' specific structural features. Additionally, the structural superposition between each *T. brucei* Hsp83 complex to each other showed that each compound induced specific structural rearrangements because superposition of non-crystallographic symmetry (NCS) related molecules revealed lower r.m.s.d values than those obtained when superposing molecules with different inhibitors bound (Table 4).

**Table 4:** Structural superposition of *T. brucei* Hsp83 NTD complexes.

		Compound 1		Compound 3		Compound 4		
		A	B	A	B	A	B	C
**Compound** 1	A	―						
	B	*0.73* *	―					
**Compound** 3	A	1.13	0.75	―				
	B	1.04	0.96	*0.53*	―			
**Compound** 4	A	1.13	0.85	0.97	1.13	―		
	B	0.97	0.76	0.88	1.02	*0.64*	―	
	C	0.99	0.77	0.92	1.04	*0.64*	*0.37*	―

(^*^) Root mean square deviation (r.m.s.d.) values in Å for all atoms superposition (residues 1 to 206). R.m.s.d. between non-crystallographic symmetry (NCS) mates is shown in italic.

### Structural analysis of TbHsp83 NTD complexes

#### *Tb*Hsp83-compound 1 complex

**Figure 5:**
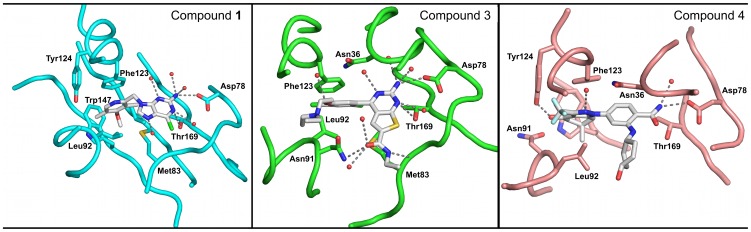
Complex structures of *T. brucei* Hsp83 and inhibitors. Protein-ligand interactions are depicted between the inhibitory compound and protein residues, indicated as sticks, the protein backbone is shown as a Cα trace. The structures are colored as follows, compound **1** complex – cyan; thienopyrimidine derivative compound **3** – green; benzamidine derivative compound **4** – brown.

**Figure 6:**
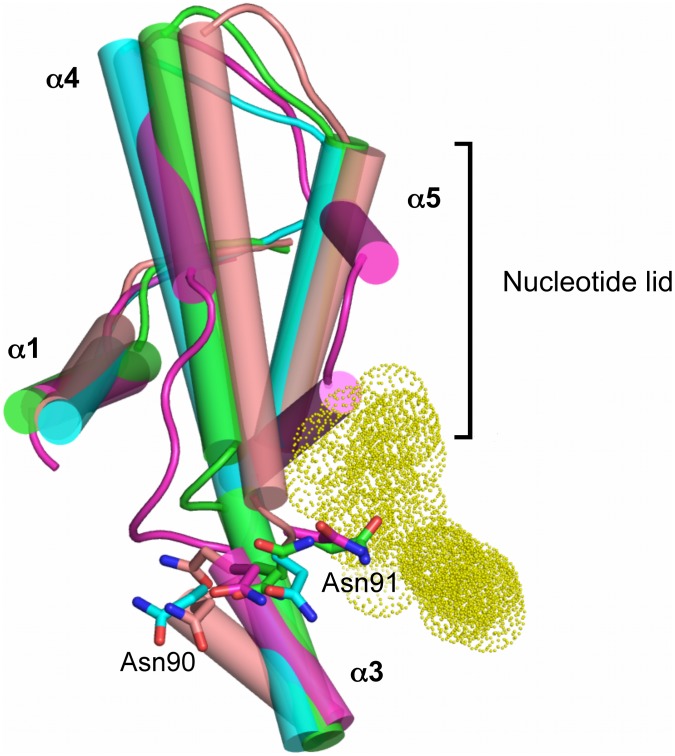
*T. brucei* Hsp83 NTD structural changes. Cartoon representation of α-helices 1, 3 and 4, and the lid region from the Hsp83 in complex with different compounds (compound **1** complex – cyan; thienopyrimidine derivative compound **3** – green; benzamidine derivative compound **4** – brown; and *L. major* with AMPPNP – magenta). Side chains of residues Asn90 and Asn91 are indicated in sticks, also the nucleotide is indicated as yellow dots to illustrate the location of binding site.

Compound **1** (commonly known as BIIB021) is a rationally designed Hsp90 inhibitor derived from a purine backbone [22]. It is composed of a modified halogenated purine moiety linked to a pyrimidyl ring (Fig. 1). The compound was slightly bent by *T. brucei* Hsp83 NTD to adopt an angle of ∼100° between the planes of the purine ring and pyrimidyl rings (Fig. 5). The purine moiety showed a hydrogen-bonding pattern already seen in the adenosine-binding pocket in other Hsp90 structures in complex with ADP or ATP mimetics (Fig. 4). The N6 (adenosine numbering scheme) was hydrogen-bonded to the δO1 of Asp78 and N1 to the γO of Thr169. Conversely, the pyridyl group occupied a modified phosphate binding pocket not seen in the nucleotide bound structures and dominated by non-polar interactions, including an aromatic π-stacking interaction between the pyridyl ring and the side chain of Phe123 (Fig. 4 & 5). The modified phosphate-binding pocket was composed of the following residues Met83, Leu92, Phe123, Tyr124, Val135 and Trp147. The elongation of helix-3 at its C-terminus joining with helix-4 into a single helix, created this modified pocket. Two residues, Asn90 and Asn91, shifted significantly their position by over 3 Å in their Cα atoms, when compared to the other *Tb*Hsp83 NTD complexes with inhibitors (Fig. 6). This movement displaced Leu92 outwards. Other regions that shifted to create pocket 2 were around residues Val135 and Trp147 (Fig. 4 & 5). These residues were in the β-sheet floor of the ATP binding site, in strands 4 and 5 that also moved outwards to accommodate the pyridyl ring. Identical structural rearrangements have been observed in the recently deposited complex between human αHsp90 NTD with compound **1** (PDB 3QDD). The r.m.d.s between the human and *Trypanosoma* complexes is 0.67 Å for a Cα superposition. More specifically, compound **1** adopts the same conformation and interacts with equivalent residues in both structures (Fig. 3A and S7). The similar structural changes correlate with our DSF assay data – compound **1** stabilized both the human and parasite proteins (Table 3 and Fig. 2A). Furthermore, they highlight the plasticity of the ATP binding domain of Hsp90 and how the conformational changes are ligand induced.

#### *Tb*Hsp83-compound 3 complex

The thienopyrimidine derivative compound **3** is a very potent human Hsp90 inhibitor identified by means of a fragment-based approach to generate novel chemical scaffolds [24]. Based on our assays, this is also a potent inhibitor of *Tb*Hsp83. The molecule is composed of a central di-halogenated phenyl ring, linked to a thienopyrimidine moiety at its position 1 and an alkyl group at position 5 (Fig. 1). The thienopyrimidine moiety occupies pocket 1, in a similar arrangement as the purine moiety of compound **1**. But its carboxamide modification establishes additional hydrogen bonds with the enzyme; a direct one with residues Gly82 and two water-mediated with residues Lys44 and Asn91. The additional interactions extend the boundaries of this pocket outside the strict adenosine-binding site. But unlike compound **1** and the benzamide derivative compound **4**, it shares several contacts with ATP, like Asn91 a hydrogen-bonding partner of the ribose moiety (Fig. 4). Additional common residues include, Asn36, Gly120 and Phe124 positioned to stabilize the γ-phosphate of ATP. Water molecules mediate the interactions between these protein residues and the thienopyrimidine derivative, distinct from the phosphonucleotide where they contact directly the phosphate group. The halogenated aromatic ring is positioned against the protein at the entrance of pocket 2, with only a chlorine atom occupying it. Its aliphatic diethylamino-ethanol substituent is mostly solvent exposed and its interaction with the chaperone was water-mediated hydrogen bonds, to Asn36 and Phe123. The position of Asn91 was different when compared to the other two complexes, in this case the side chain makes a direct contact with the small molecule in a conformation expected in the complex of Hsp90 with phosphonucleotides. A major structural characteristic of this complex is the modification of helices 3 and 4, each shortened by a couple of residues. The unwinding of helix 3 and 4 pushes the position of the latter closer to helix 1, shifting its position by over 1 Å (Fig. 6).

#### *Tb*Hsp83-compound 4 complex

**Figure 7:**
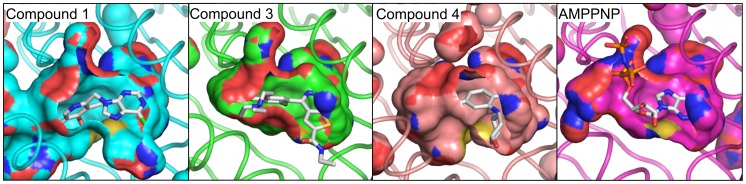
*T. brucei* Hsp83 nucleotide binding pocket. Surface representation in the NTD binding site for the *Trypanosoma* complex structures (compound **1** complex – cyan; thienopyrimidine derivative compound **3** – green; benzamidine derivative compound **4** – brown). The nucleotide binding pocket of *L. major* (PDB code 3H80 – Magenta) is shown for comparison purposes.

The benzamide derived compound **4** was identified through a chemogenomics approach coupled to lead optimization to produce a highly selective and orally active Hsp90 inhibitor for cancer treatment [48]. As seen in the compound **3** complex, the benzamide ring occupies the purine pocket in the same way as the natural nucleotide and establishes similar polar contacts as the other two *T. brucei* Hsp83 inhibitors. The modified dihydroindazolone group occupies the pocket 2 but unlike the previous compounds a hydrogen bond was observed with Tyr124 (Fig. 4). In addition to the polar interaction, the presence of an electronegative trifluoromethyl group creates a smaller phosphate binding pocket resulting in a tight packing between the compound and the protein, when compared with that seen in the *Tb*Hsp83-compound 1 NTD complex (Fig. 7). Other polar interactions through a water molecule were observed between the pyrazole ring and residues Asn36 and Gly120, similar to those observed with the thienopyrimidine derivative complex. Both compounds appear to exploit this water molecule that mimics the position of one of the phosphate groups of ATP. The largest structural rearrangement observed in this complex was the flipping in of Asn91 side chain that created a disruption at beginning of the helix-4. The new position of Asn91 side chain was stabilized by a polar interaction with the indole nitrogen of Trp147. This interaction is an important step to narrowing pocket 2. The tilted position of helix-4 moving towards the binding pocket also affected the secondary structure at helix-4 C-terminus changing from compact turns observed in the other two complexes to extended β-bridges. Overall these structural rearrangements create a closed pocket that occludes most of the inhibitor from the solvent (Fig. 4 & 6). All complex *Tb*Hsp83-inhibitor complex crystallographic structures displayed unique structural features emphasizing the malleability of the chaperone nucleotide-binding domain.

## Discussion

From our results, the ATPase activity of the *Trypanosoma* chaperone is within the range of measured activities for this class of enzymes, which are characterized by a rather low turnover and high *K*_m_ values (Table 2) [17]. In comparison with previously reported enzymatic constants the *T. brucei* chaperone has a slightly higher affinity for its substrate [7], [40], [41]. Lower *K*_m_ values have also been reported for other Hsp90s, albeit at a lower incubation temperature 25°C [17]. In addition to the low affinity for its substrate, Hsp90 and its homologues tend to have slow turnover rates, ranging from 0.0001 to 0.02 s^−1^ [17]. Nonetheless, the *T. brucei* chaperone has a ten-fold higher turnover rate together with a higher affinity, which makes for a more efficient ATPase. There is no clear explanation for this enhanced ATPase activity but a similar observation has also been made for *Plasmodium falciparum* Hsp90 [7], another human parasite. This is an interesting trend to be evaluated as new activity reports from parasitic chaperones become available. A tantalizing hypothesis is that parasites might rely more heavily on their Hsp90 chaperones to maintain cellular homeostasis by stabilizing key cell regulators under hostile conditions. An observation that would be consistent with human Hsp90's pivotal role in some forms of cancer [49] (leading to its label as the Achilles' heel of malignant cells), a role that could also be exploited to treat parasitic infections [18]. Finally, a role has been determined for this chaperone in *Candida albicans* in the resistant phenotypes against commonly used antifungal drugs, then a plausible hypothesis could be that the parasitic hyperactive Hsp90 could facilitate the emergence of drug resistant parasites [50], [51].

Previous studies have determined the sensitivity of several parasitic chaperones to known inhibitors, such as geldanamycin and radicicol, and their deleterious effects on parasite growth [6], [7], [8], [9], [10], [52], [53]. To identify and characterize inhibitors of *Tb*Hsp83, we used the DSF technique to screen a collection of near 40 compounds described in the literature as known or putative Hsp90 binders. The bulk of the collection includes ATP-competitive inhibitors predicted to bind the N-terminal region but also several putative C-terminal inhibitors. Initially, our DSF assays were performed with both full-length proteins and individual domains and two observations were derived from these experiments.

In the case of the human Hsp90s, there appears to be a similar Δ*T*_m_ for both the N-terminal domain and the full length protein, whilst in the case of the *T. brucei* Hsp83, the Δ*T*_m_ appears to be greater for the N-terminal domain, with a significantly weaker shift for the full length protein. The *T. brucei* C-terminal domain, the middle domain and the combined C-terminal & middle domain were also tested and did not show any significant Δ*T*_m_, as would be expected for compounds which bind in the N-terminal ATP binding site (Fig. S2). Taken together, these observations suggest (i) the compounds interact primarily with the N-terminal ATPase domain, as most of the them are known to do with other Hps90 proteins; (ii) the DSF assay is an effective technique for identifying potential Hsp90 inhibitors; (iii) we can focus on the N-terminal ATPase domain for characterizing and optimizing inhibitors of *Tb*Hsp83.

We observed an inverse relationship (R^2^ = 0.88) existed between ITC-derived *K*_d_ values and DSF-derived Δ*T*_m_ values, with the caveat that some of the ITC reported affinities might be under or overestimated affecting the correlation between the two assays (Fig. S5). Such a relationship has been reported for other proteins such as protein kinases [54] and bromodomains [55], but with even higher correlation coefficients. The observed correlation would likely be greater if the tested compounds were structurally related because the correlation is stronger for compounds sharing a common binding mode. By virtue of its operating principles, the DSF method is particularly sensitive to entropy-driven binding [56]. Therefore, when contributions of enthalpy and entropy are different, change in Δ*T*_m_ may not correspond to *K*_d_ [57], [58], [59]. The lower detection limit of our thermal shift assay is in the micromolar range when using a compound concentration of 25 µM (e.g., compound 2 with Δ*T*_m_ = 0.8°C and *K*_d_ = 9.3 µM.). The observed limit explains the absence of hits for the C-terminal domain since they have been known to bind with low affinity (10^−3^ to 10^−6^ M range). Nonetheless, our approach has been successful in identifying high affinity binders by screening a narrow set of Hsp90 inhibitors rather than a broad diverse library. Our results support the DSF assay as a suitable technique to screen chemical libraries originally designed against human targets, as we screened compounds originally designed as anti-human Hsp90 inhibitors.

The three-dimensional structure of the NTD of *T. brucei* Hsp83 in complex with three different inhibitors showed the large degree of structural flexibility of the chaperone. As was previously described, the nucleotide-binding site can be divided into the adenosine and the phosphate binding pockets [25]. All the inhibitors described here occupy both pockets but differ in how they expand or modify these binding cavities [60], [61], [62]. All of the inhibitor scaffolds recapitulate the adenosine mode of binding to the chaperone (Fig. 4 & 5). Previous structures have established that the base is recognized by its Watson-Crick face generating two hydrogen bonds with the NTD; one is a water-mediated interaction while the other one is between the base amino group and the side chain of a well conserved aspartic acid residue (Asp78 in *T. brucei*). All of the inhibitors described here preserve these interactions, including the water mediated one. Interestingly, the nucleotide-binding site of the NTD is highly conserved among different Hsp90 family members, with the exception of Ile171 (located in the region of sequence divergence highlighted in orange in Fig. S1) being a moderately variable position located at the bottom of the adenosine-binding pocket (Fig. S8). This side chain is in contact with residue Leu34 and indirectly with Ile35, a pair of residues directly implicated in resistance to the natural product radicicol [46]. This observation could rationalize the significant differences measured by our DSF assay between the *Tb*Hsp83 and the human α and β Hsp90s with respect to radicicol. It also highlights the large impact that small sequence differences could have in the chemical sensitivity of the parasitic chaperone.

The thienopyrimidine derivative, namely compound **3**, was the only compound to establish charged contacts in the periphery of the adenosine binding pocket with residues involved in the interaction with the sugar moiety of the phosphonucleotide, such as Asn91, while the other two compounds engaged these residues in non-polar interactions (Fig. 4 & 5). As a result, this region located at the end of helix-3 between residues 87 to 93 showed the largest structural differences among the three complex structures with values exceeding 4 Å r.m.s.d. for the main chain atoms of residues Asn91 and Asn92 (Fig. 6). This structural rearrangement tilted the position of helix 4, a key structural element in the NTD, because together with helix 5 it composes the lid region. The closing of the lid over the ATP during the catalytic cycle is a preliminary step also linked to the dimerization of the NTDs, as it allows the movement of helix 1 and facilitates the exchange of the N-terminal β-strands among interacting NTDs. On the basis of the large structural movements displayed during the ATPase cycle of Hsp90, we propose the region located at the end of helix 3 plays an important role as a sensor for the binding of nucleotides a function facilitated by its structural plasticity and highlighted by recent NMR studies of the Hsp90 [63]. In our case, the region of structural flexibility allows the NTD to accommodate a diverse set of compounds generating unique binding regions for each one. The uniqueness of the conformations induced by each compound to the region encompassed by residues 87 to 93 has extensive repercussions in the lid position. Each complex structure showed a unique open conformation of the lid region (Fig. 6) with the subsequent impact on the phosphate-binding pocket (Fig. 7). This structural flexibility was already described for the NTD when binding both substrate and inhibitors and two main conclusions can be drawn from these observations. First, due to the large structural rearrangements involved upon inhibitor association, the contribution from regions other than the nucleotide-binding pocket can be quite substantial and hard to predict by analyzing the structure alone. Second, the cellular effects of Hsp90 inhibitors are related to the conformational limitations imposed on the chaperone, affecting co-chaperone binding and long-range coupled motions observed between the NTD and CTD domains [64].

Compound **5** preferentially binds *Tb*Hsp83 with high affinity but not human Hsp90, but did not yield diffracting crystals in our attempts. Therefore, we are unable to provide a definitive structural explanation for this benzamide's selective behavior. We also cannot utilize existing human Hsp90 structures and our *Tb*Hsp83 structures for this purpose because, as explained above, the chaperone's flexibility allows it to be reshaped differently by different classes of inhibitors. The chemical selectivity of compound **5** suggests that it uniquely recognizes the difference between the flexibility the parasite and human chaperones. Remarkably, the differentiation is a product of one or more of the three short regions of sequence divergence shown in Fig. S1.

This study presents a detailed structural and chemical characterization of *Tb*Hsp83 as a potential drug target against African trypanosomiasis a fatal neglected disease. We have identified a chemical scaffold with a preference for the *Trypanosoma* chaperone over its host counterpart. Furthermore, the assayed compounds were able to inhibit the growth of the parasite *in vitro* in a manner that correlate strongly to their binding affinities to *Tb*Hsp83, validating this parasite chaperone as a potential target to combat sleeping sickness. Target validation and early lead identification represent the initial stages of a potential drug development program. Furthermore, in the Hsp90 case, there is the possibility of taking advantage of the much more advanced anti-Hsp90 programs in the cancer field. We believe that tapping into other therapeutics areas such as cancer might have a great value in the development of drugs against neglected tropical diseases.

Generally speaking, a potential weakness in repurposing compound libraries designed against human targets to search for anti-parasitic compounds is that the starting chemical points are not selective. What is important, however, is to show the possibility of differentiating structure activity relationship between the human and parasite enzymes. In spite of the high degree of sequence identity with the human homologue, we have found such a chemical differentiator for the NTD of *Tb*Hsp83 in compound **5** and a number of other analogues. More research is required to substantiate and elaborate on our hypothesis that this selectivity is a result of differences in the flexibility of the human and parasite chaperones.

## Supporting Information

Figure S1 Alignment of sequences of N-terminal ATPase domains from *Tb*Hsp83, *Lm*Hsp83, Hsp90 and Hsp90, with 3 regions of sequence divergence highlighted.
(TIFF)

Figure S2 DSF assay results represented as a heat map for the *Tb*Hsp83 (full length and domains) and human Hsp90 isoforms α and β. Bibliographic references for each compound used are indicated in parentheses.
(TIFF)

Figure S3 ATPase activity of *Tb*Hsp83. A graph of enzyme velocity in µmoles of inorganic phosphate produced per second against substrate (ATP) concentration.
(TIFF)

Figure S4 ITC binding data of *Tb*Hsp83 against several anti-Hsp90 compounds.
(TIFF)

Figure S5 Graphs of DSF derived Δ*T_m_* against ITC derived dissociation constant (A) and EC_50_ from parasite growth inhibition assay (B). A regression line is depicted.
(TIFF)

Figure S6 Stereo diagram of simple difference simulating-annealing omit maps for the three compounds crystallized with *Tb*Hsp83. The maps are contoured at +3σ and −3σ colored green/purple and red respectively.
(TIFF)

Figure S7 Superposition between human (yellow) and *T. brucei* (cyan; chains A and B) Hsp90 NTD in complex with Compound 1.
(TIFF)

Figure S8 Sequence differences between *Tb*Hsp83 and human Hsp90s isoforms α and β. Surface representation of the N-terminal domain of *Tb*Hsp83 color-coded according to sequence conservation, blue – conserved and yellow – variable residues.
(TIFF)

Table S1 List of all tested compounds and their origin, commercial (C) or synthesized (S).
(TIFF)
