# Supplementary figures and images for: Exploring the Trypanosoma brucei Hsp83 Potential as a Target for Structure Guided Drug Design
Source: PLoS Negl Trop Dis. 2013 Oct 17;7(10):e2492. doi: 10.1371/journal.pntd.0002492 (PMC3798429; doi:10.1371/journal.pntd.0002492)

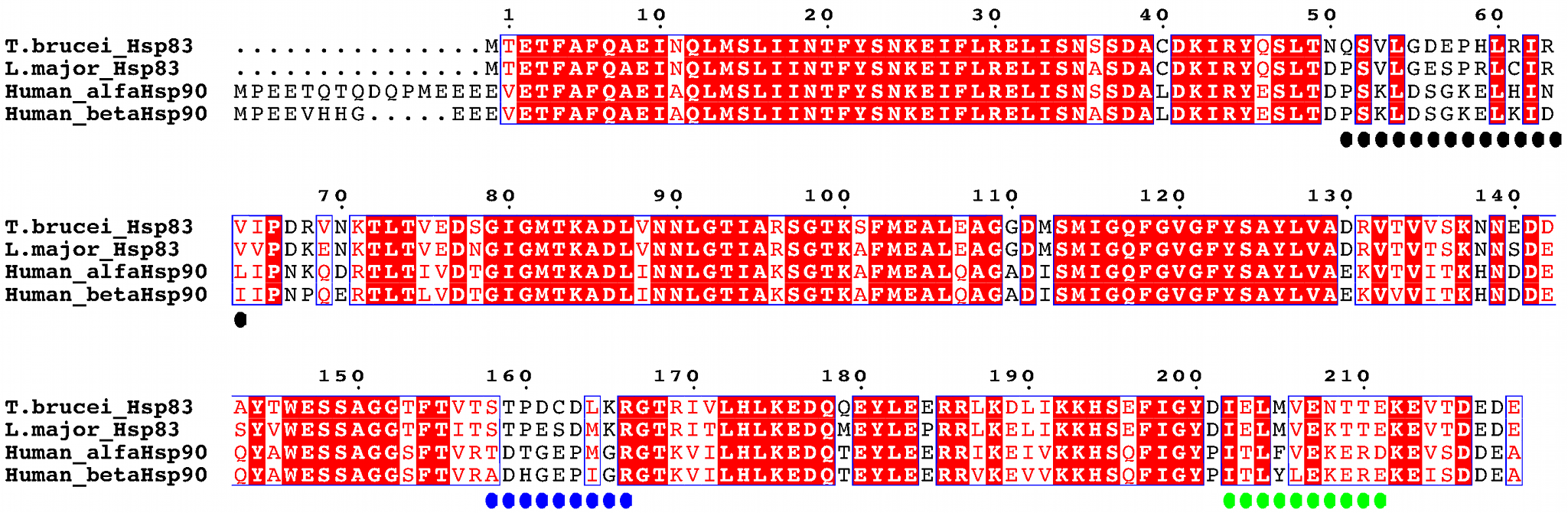

Supplement: Figure S1 — Alignment of sequences of N-terminal ATPase domains from TbHsp83, LmHsp83, Hsp90 and Hsp90, with 3 regions of sequence divergence highlighted. (TIFF) [file pntd.0002492.s001.tiff]

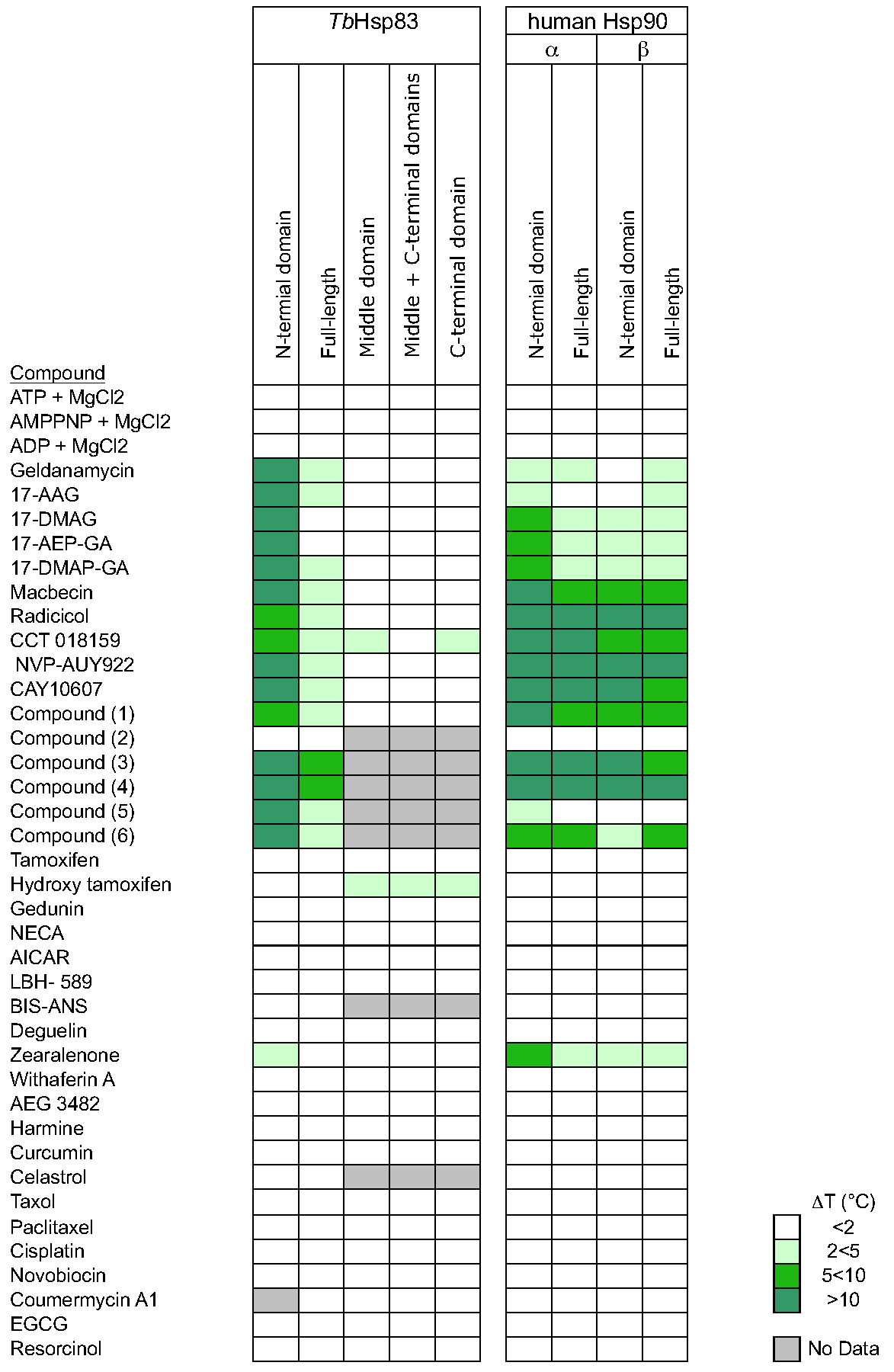

Supplement: Figure S2 — DSF assay results represented as a heat map for the TbHsp83 (full length and domains) and human Hsp90 isoforms α and β. Bibliographic references for each compound used are indicated in parentheses. (TIFF) [file pntd.0002492.s002.tiff]

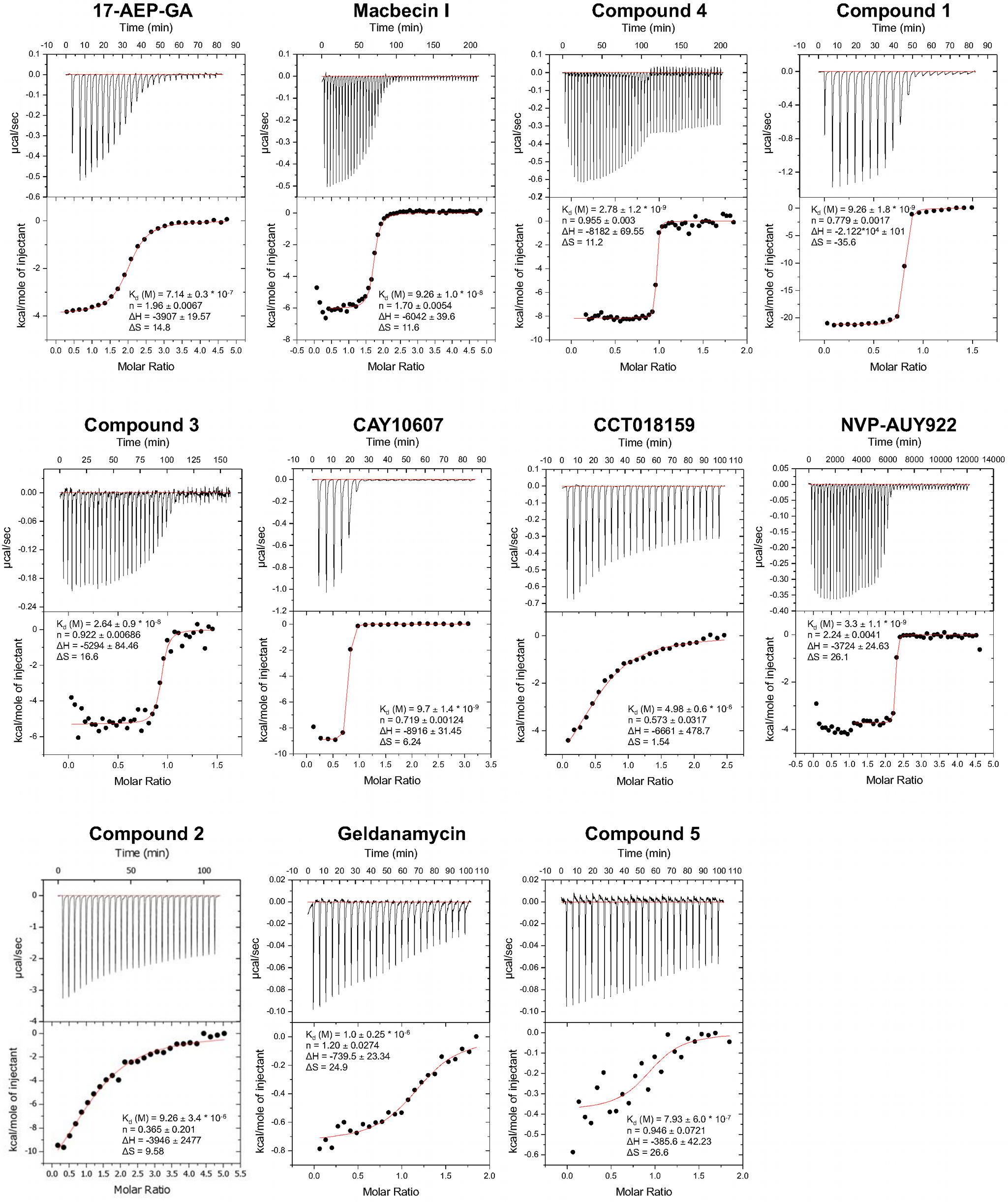

Supplement: Figure S4 — ITC binding data of TbHsp83 against several anti-Hsp90 compounds. (TIFF) [file pntd.0002492.s004.tiff]

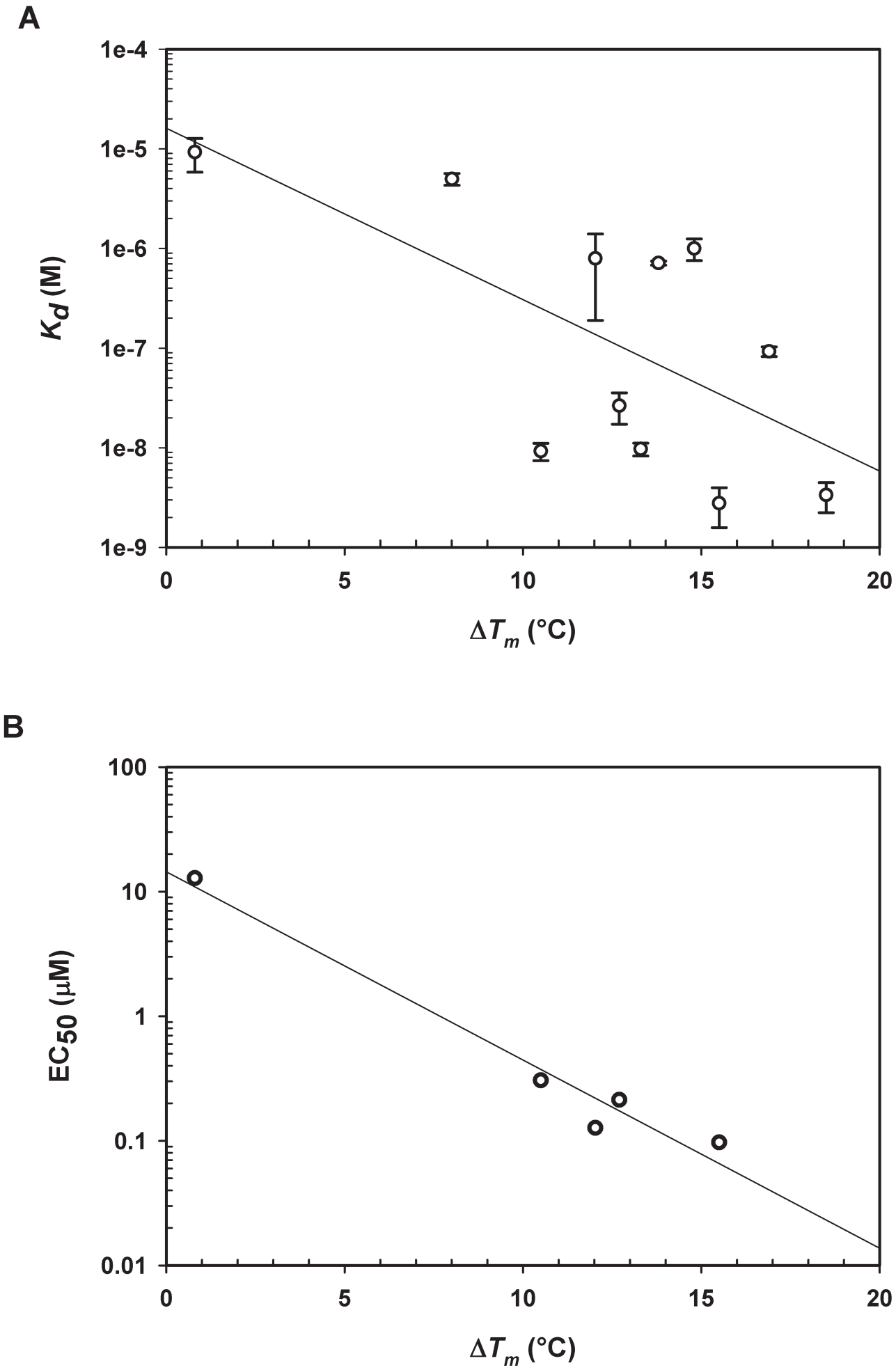

Supplement: Figure S5 — Graphs of DSF derived ΔTm against ITC derived dissociation constant (A) and EC50 from parasite growth inhibition assay (B). A regression line is depicted. (TIFF) [file pntd.0002492.s005.tiff]

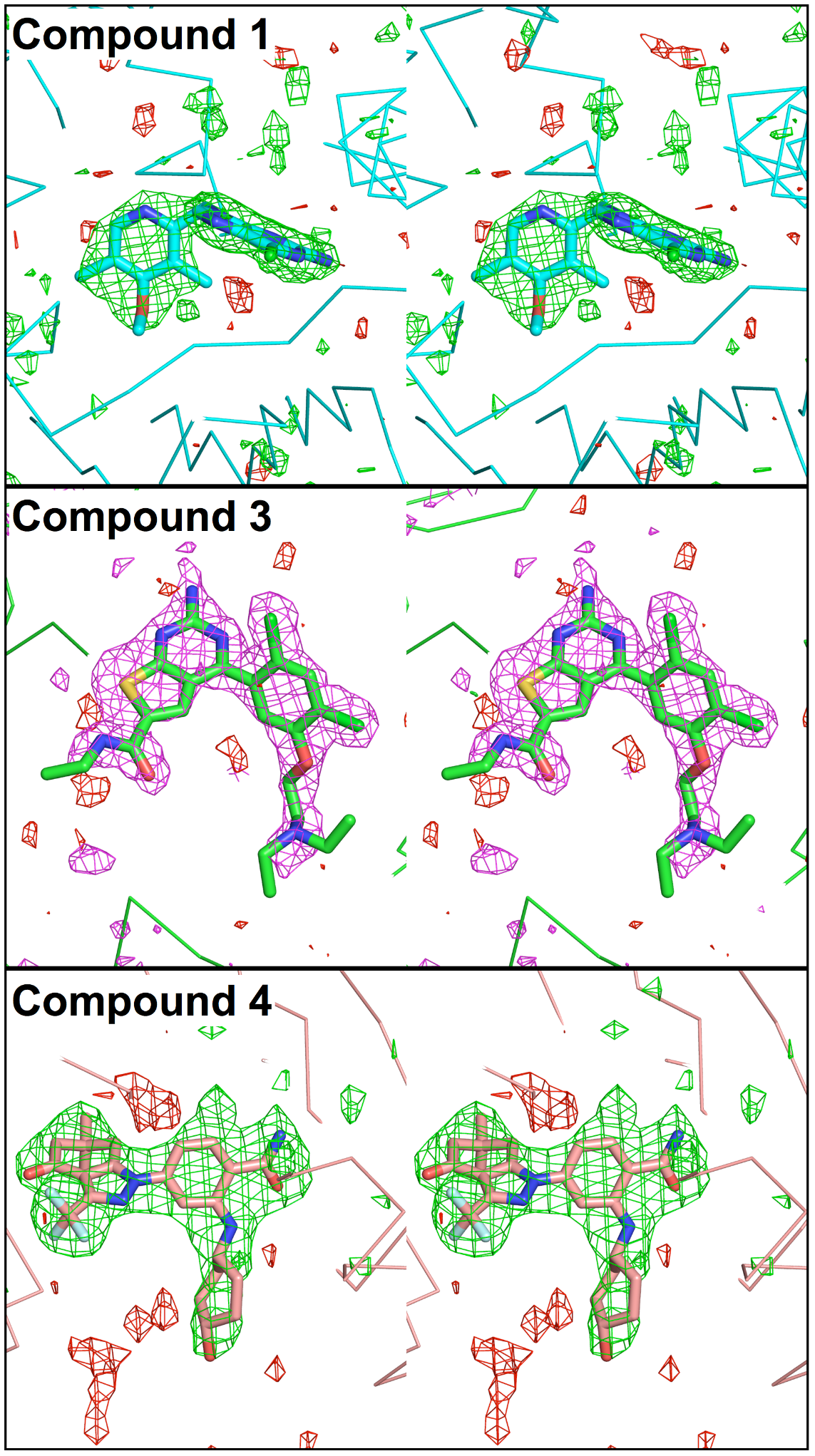

Supplement: Figure S6 — Stereo diagram of simple difference simulating-annealing omit maps for the three compounds crystallized with TbHsp83. The maps are contoured at +3σ and −3σ colored green/purple and red respectively. (TIFF) [file pntd.0002492.s006.tiff]

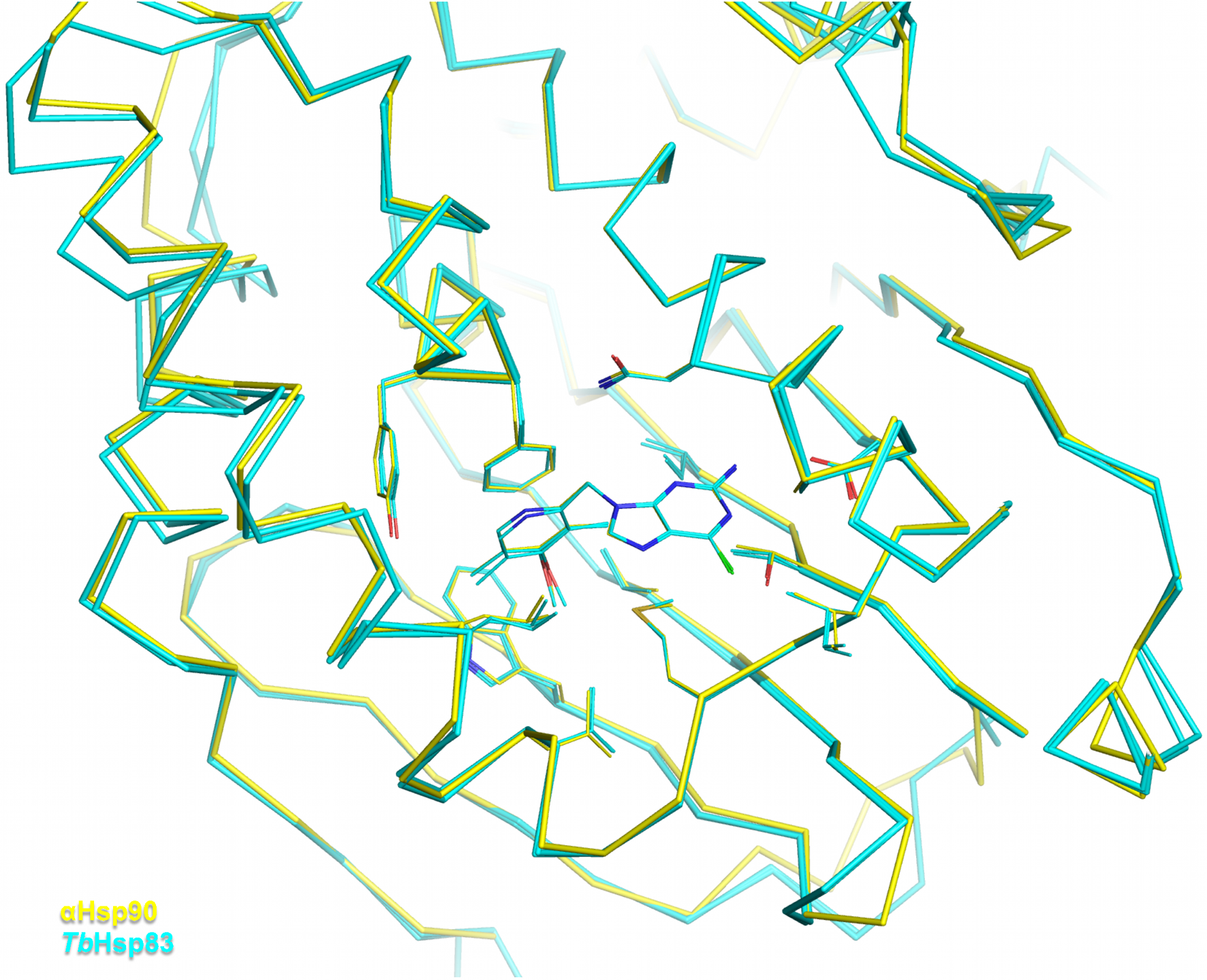

Supplement: Figure S7 — Superposition between human (yellow) and T. brucei (cyan; chains A and B) Hsp90 NTD in complex with Compound 1. (TIFF) [file pntd.0002492.s007.tiff]

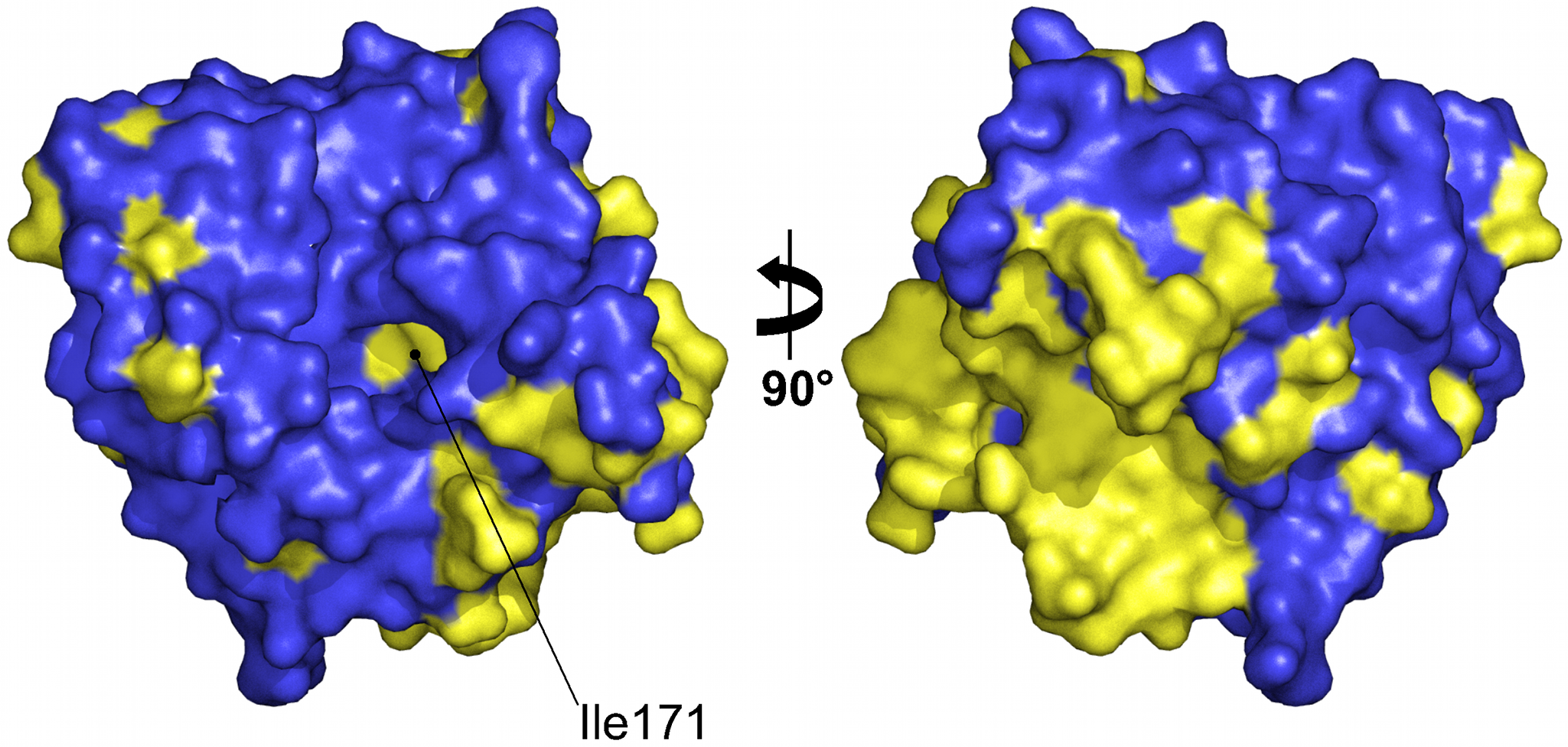

Supplement: Figure S8 — Sequence differences between TbHsp83 and human Hsp90s isoforms α and β. Surface representation of the N-terminal domain of TbHsp83 color-coded according to sequence conservation, blue – conserved and yellow – variable residues. (TIFF) [file pntd.0002492.s008.tiff]
